# Impact of Geographic Location on Diagnosis and Initial Management of Takayasu Arteritis: A Tale of Two Cohorts from Italy and India

**DOI:** 10.3390/diagnostics12123102

**Published:** 2022-12-09

**Authors:** Durga Prasanna Misra, Alessandro Tomelleri, Upendra Rathore, Giovanni Benanti, Kritika Singh, Manas Ranjan Behera, Neeraj Jain, Manish Ora, Dharmendra Singh Bhadauria, Sanjay Gambhir, Sudeep Kumar, Elena Baldissera, Vikas Agarwal, Corrado Campochiaro, Lorenzo Dagna

**Affiliations:** 1 Department of Clinical Immunology and Rheumatology, Sanjay Gandhi Postgraduate Institute of Medical Sciences (SGPGIMS), Lucknow 226014, India; 2 Unit of Immunology, Rheumatology, Allergy and Rare Diseases (UnIRAR), IRCCS San Raffaele Hospital, Vita-Salute San Raffaele University, 20132 Milan, Italy; 3 Department of Nephrology, Sanjay Gandhi Postgraduate Institute of Medical Sciences (SGPGIMS), Lucknow 226014, India; 4 Department of Radiodiagnosis, Sanjay Gandhi Postgraduate Institute of Medical Sciences (SGPGIMS), Lucknow 226014, India; 5 Department of Nuclear Medicine, Sanjay Gandhi Postgraduate Institute of Medical Sciences (SGPGIMS), Lucknow 226014, India; 6 Department of Cardiology, Sanjay Gandhi Postgraduate Institute of Medical Sciences (SGPGIMS), Lucknow 226014, India

**Keywords:** Takayasu arteritis, aortic arch syndromes, arteritis, systemic vasculitis, healthcare disparities, Italy, India

## Abstract

The present study compares disease characteristics, imaging modalities used, and patterns of treatment in two large cohorts of Takayasu arteritis (TAK) from Italy and India. Clinic files were retrospectively reviewed to retrieve information about initial choices of vascular imaging and immunosuppressive therapies. Unpaired t-tests compared means, and proportions were compared using Fisher’s exact test or Chi square test [Odds ratios (OR) with 95% confidence intervals (95%CI) calculated where appropriate]. The cohorts comprised 318 patients [Italy (*n* = 127), India (*n* = 191)] with similar delays to diagnosis. Ultrasound (OR Italy vs. India 9.25, 95%CI 5.02–17.07) was more frequently used in Italy and CT angiography in India (OR 0.32, 95%CI 0.20–0.51). Corticosteroid use was more prevalent and for longer duration in Italy. TAK from Italy had been more often treated with methotrexate, leflunomide or azathioprine, as opposed to tacrolimus in TAK from India (*p* < 0.05). Biologic or targeted synthetic disease-modifying agents were almost exclusively used in Italy. Survival on first immunosuppressive agent was longer from Italy than from India (log rank test *p* value 0.041). Considerable differences in the choice of initial vascular imaging modality and therapies for TAK from Italy and India could relate to prevalent socio-economic disparities. These should be considered while developing treatment recommendations for TAK.

## 1. Introduction

Takayasu arteritis (TAK) is a granulomatous large vessel vasculitis (LVV) that has a propensity to affect younger females [1]. Involvement of the aorta and its branches in TAK presents in several ways, ranging from clinically silent incidentally detected pulse loss to stroke or myocardial infarction. Overall, TAK is a rare disease that is more common in Asian countries than in Europe or North America [2,3]. 

Disease activity in TAK is challenging to assess due to the inconsistent association of erythrocyte sedimentation rate (ESR) and C-reactive protein (CRP) levels with vascular inflammation [4]. Indices such as the Disease Extent Index in TAK (DEI.TAK), Indian TAK Clinical Activity Score (ITAS2010), and the National Institutes of Health (NIH) disease activity criteria are adjuncts to assess clinical disease activity in TAK, albeit demonstrating inconsistent concordance with physician global assessment [5,6,7]. Unlike in giant cell arteritis (GCA), the arterial territories involved in TAK are difficult to evaluate for histopathological evidence of active disease except during vascular bypass procedures [2,3]. In the past, imaging of the arterial tree in TAK required invasive modalities such as conventional angiography. Now, it predominantly relies on computerized tomographic angiography (CTA) and magnetic resonance angiography (MRA). The use of 18-fluorodeoxyglucose (18-FDG) positron emission tomography (PET), combined with CT or magnetic resonance imaging (MRI) for anatomical evaluation, enables in-vivo visualization of metabolic activity in the large vessels (which might indicate active vascular inflammation) [2,3,8]. 

High-dose glucocorticoids are highly effective in active TAK; however, relapses inevitably occur when tapered or withdrawn [9]. To maintain clinical remission, disease-modifying anti-rheumatic drugs (DMARDs) are commonly used [10]. Generally, conventional-synthetic DMARDs (csDMARDs) such as methotrexate, azathioprine, or mycophenolate are used as first-line treatment. Biologic DMARDs (bDMARDs) or, more recently, targeted synthetic DMARDs (tsDMARDs) are commonly initiated in patients with relapses or disease progression despite being on csDMARDs. However, since no randomized controlled trial has met its primary end-points in TAK, the use of glucocorticoids and DMARDs is based predominantly on observational data [2,10,11,12,13,14,15,16,17,18,19,20,21,22,23,24,25,26]. For those arterial territories with critical vascular stenoses resulting in end-organ damage (such as limb claudication, carotid artery occlusion, myocardial infarction, or refractory hypertension), the restoration of vascular flow by open surgical bypass grafts or endovascular procedures might represent the only solution [2,3]. Mortality in patients with TAK can result from end-stage heart failure, uncontrolled hypertension resulting in hemorrhagic stroke or chronic kidney disease, critical vascular occlusion causing ischemic stroke, myocardial infarction or bowel gangrene, or from infections (most commonly secondary to treatment-related immunosuppression) [27]. 

Few recommendations for the diagnosis and management of TAK are available, and these are mostly from Europe and North America [28,29]. While large cohorts of TAK have now been described from most parts of the world, differences in the presentation, the angiographic extent of disease, diagnostic modalities, and management between different geographic regions have not been systematically studied. In this observational cohort study, we compare two large cohorts of TAK from Italy (Europe) and India (Asia) to understand differences in the diagnosis and management of this uncommon disease. Understanding such differences might facilitate the eventual development of guidelines for TAK that are relevant for patients across the globe. 

## 2. Materials and Methods

### 2.1. Patient Selection

Case files from two retrospective cohorts of patients with TAK who visited dedicated vasculitis clinics from Milan, Italy, and Lucknow, India were analyzed. Information was retrieved using common pre-designed case record forms for data extraction. Due to the nature of retrospective data retrieval without contact with individual patients, the requirement for written informed consent was waived (by the Institute Ethics Committee, SGPGIMS 2021-165-IP-EXP-40). Written informed consent was obtained from all patients enrolled in the Italian cohort (PanImmuno Protocol). Included patients fulfilled either the 1990 American College of Rheumatology classification criteria or the 2012 Chapel Hill Consensus Conference definition for TAK for those with age of onset more than 18 years [30,31], or the European Alliance of Associations for Rheumatology/Pediatric Rheumatology European Society/Pediatric Rheumatology International Trials Organization classification criteria for TAK if the age of onset was ≤18 years [32]. 

### 2.2. Clinical Characteristics and Imaging Studies

Age at cohort entry, sex, diagnostic delay (from the first symptom to diagnosis), and prevalent comorbid conditions (diabetes mellitus, smoking, dyslipidemia, cancer, concomitant immune-mediated inflammatory diseases, or others) recorded at cohort entry were noted. Baseline disease activity status indicated by DEI.TAK, ITAS2010, and physician global assessment (active or inactive) were recorded [6,7]. Any recorded mortality during follow-up was also noted. Clinical features, baseline inflammatory markers (ESR in mm/hour, CRP in mg/L), and the duration of follow-up data available were evaluated. Imaging modalities used at the initial assessment [CTA, MRA, PET-CT, PET-MRI, conventional angiography/digital subtraction angiography, and color doppler ultrasonography (CDUS)] were noted (a single patient could have utilized more than one imaging modality). Individual vessels involved at initial presentation and the angiographic classification of the disease as per Hata’s classification [I, IIa, IIb, III, IV, V, with or without the involvement of pulmonary arteries (P+) or coronary arteries (C+)] were recorded [33]. 

### 2.3. Drug Treatments Received

Baseline treatment with glucocorticoids, the proportion of total patients receiving intravenous methylprednisolone, the initial dose of daily glucocorticoids (in prednisolone equivalent doses), duration of glucocorticoid therapy (in months), the proportion of patients who were continuing glucocorticoids at the last follow-up, and the percentage reduction in prednisolone dose between first and last visits were analyzed. Concerning steroid-sparing agents, the proportion of patients treated with a particular DMARD, proportion of patients initiated on a DMARD who were continuing at the last follow-up visit, and duration of treatment with a particular DMARD (in months) were calculated for individual conventional, biologic, or targeted synthetic DMARDs. The mean number of csDMARDs and b/tsDMARDs per individual patient was calculated. Whenever the use of medical therapies between the two cohorts was compared, differences in the proportions of individuals continuing the medications at the last visit were statistically analyzed only for those medications which had been used in both cohorts. The proportion of patients treated with anti-hypertensive medications, the number of anti-hypertensive medications required at baseline, and the proportions of patients receiving aspirin, P2Y12 inhibitors, or statins were also recorded. 

### 2.4. Procedures and Infections on Follow-Up

The number of open (surgical bypass grafts, nephrectomy for refractory hypertension, or laparotomy for bowel ischemia) or endovascular procedures related to TAK undergone by the patients and the proportion of patients undergoing such procedures were recorded. The timing of procedures (before or after diagnosis of TAK) was recorded. The number of infections requiring hospitalization or resulting in death in each cohort, the number of patients developing such infections, the dose of prednisolone, and the usage of DMARDs at the time of such infection were recorded.

### 2.5. Statistical Analysis

A formal sample size calculation was not undertaken given the exploratory nature of the study and the rarity of TAK. The data were compared between the two cohorts using the unpaired t-test for means (with standard deviation), or by using Fisher’s exact test (if either of the numerators of the proportions was less than 5) or Chi-square test to calculate 2-sided *p*-values. Odds ratios (OR, along with 95% confidence intervals, 95% CI) for clinical features at presentation, vessels involved at presentation, Hata’s angiographic classification, and imaging modality used at the initial assessment for Italy vs. India were calculated. Wherever multiple comparisons were performed for related covariates, the *p* values were corrected for multiple testing by using the Bonferroni-Sidak correction. The duration of persistence of the first DMARD in months was compared between the two cohorts using Kaplan-Meier plot with the log-rank test and hazard ratio calculated using Cox proportional hazards. Statistical analyses were performed using Prism 9 for macOS [Version 9.3.1 (350)] and STATA 16.1 I/C (for Kaplan-Meier plot)]. *p* values ≤ 0.05 were considered statistically significant. 

## 3. Results

### 3.1. Demographic and Clinical Characteristics

**Figure 1:**
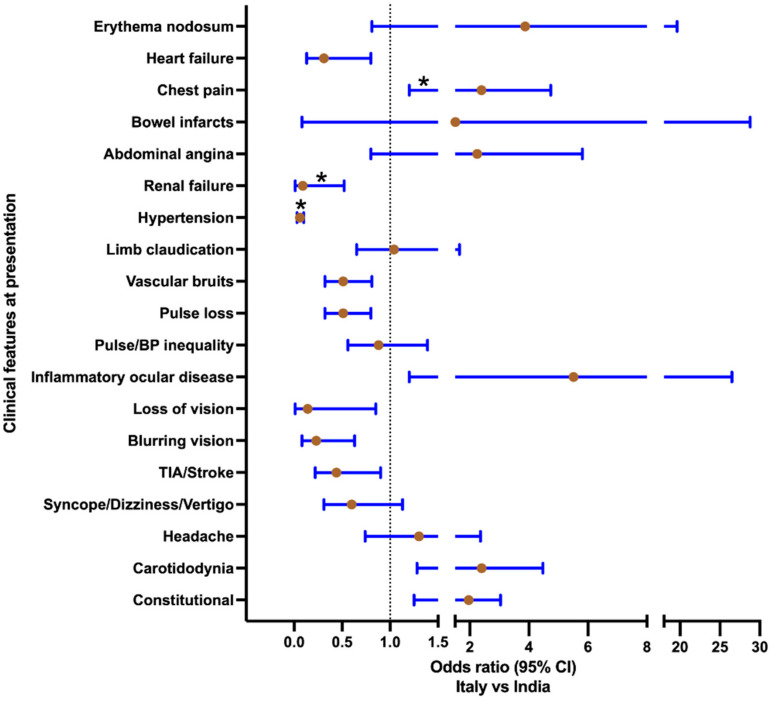
**Comparison of clinical features at presentation in patients with Takayasu arteritis from Italy and India.** Odds ratio (95% confidence intervals) presented for odds ratio for clinical features at presentation for Italy vs. India (Italy, *n* = 127; India, *n* = 191). Those comparisons marked with an asterisk were different between the two cohorts with *p* < 0.05 after correction for multiple testing.

There were 127 patients in the cohort from Italy and 191 from India (Table 1). Both cohorts had a preponderance of female patients, though there was a greater proportion of females in the cohort from Italy. Patients with TAK from India were younger at cohort entry. The delay to diagnosis (from the first symptom appearance) was similar. The cohort from Italy had a significantly longer duration of follow-up than that from India (mean, 130 months vs. 46 months). Concerning comorbid conditions at the first visit, smoking, dyslipidemia, and concomitant immune-mediated diseases were more common in the Italian cohort. A greater proportion of patients from Italy than from India had active disease at baseline as per the physician global assessment (91.3% vs. 80.1%). However, DEI.TAK and ITAS2010 at baseline were higher for patients with TAK from India than from Italy. At presentation, hypertension (OR 0.06, 95% CI 0.03–0.10) and renal failure (OR for Italy vs. India 0.09, 95% CI 0.01–0.52) were more frequent in TAK from India, whereas acute coronary syndrome was more frequent in TAK from Italy. Inflammatory markers (i.e., ESR and CRP) were higher at baseline in TAK from Italy than from India (Figure 1, Supplementary Table S1). 

### 3.2. Imaging Modalities and Vascular Involvement at the Initial Assessment

**Figure 2:**
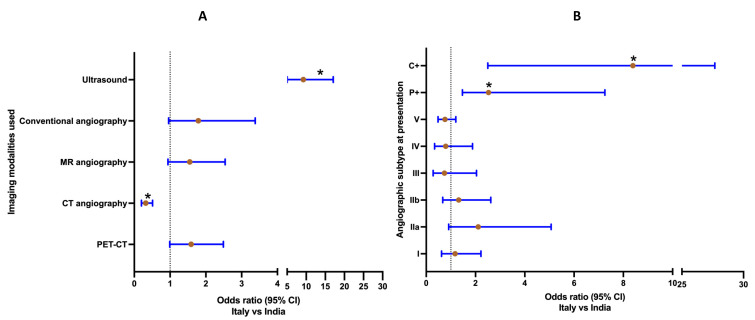
(**A**) **Comparison of imaging modalities used at initial assessment in patients with Takayasu arteritis from Italy and India**. Odds ratio (95% confidence intervals) presented for odds ratio for imaging modalities used at initial assessment for Italy vs. India (Italy, *n* = 127; India, *n* = 191). Those comparisons marked with an asterisk were different between the two cohorts with *p* < 0.05 after correction for multiple testing. (**B**) **Comparison of Hata’s angiographic classification at baseline in patients with Takayasu arteritis from Italy and India**. Odds ratio (95% confidence intervals) presented for odds ratio for Hata’s angiographic classification at baseline for Italy vs. India (Italy, *n* = 127; India, *n* = 191). Those comparisons marked with an asterisk were different between the two cohorts with *p* < 0.05 after correction for multiple testing.

CDUS (OR 9.25, 95% CI 5.02–17.07) was more frequently used at the first evaluation in the TAK cohort from Italy, whereas CT angiography was more frequently used in the TAK cohort from India (OR 0.32, 95% CI 0.20–0.51) (Figure 2A, Supplementary Table S2). Coronary arteries (OR 8.35, 95% CI 2.49–27.52), right carotid artery (OR 2.14, 95% CI 1.36–3.34), right vertebral artery (OR 6.43, 95% CI 2.52–15.55), and pulmonary artery (OR 3.22, 95% CI 1.46–7.21) were more frequently involved in the TAK cohort from Italy, whereas the renal arteries (right renal—OR 0.36, 95% CI 0.22–0.61; left renal OR 0.35, 95% CI 0.21–0.60) were more frequently involved in the TAK cohort from India (Supplementary Table S2). Angiographic subtypes as per Hata’s classification were similar in both cohorts except for a greater prevalence of coronary artery involvement and pulmonary artery involvement in the cohort from Italy (Figure 2B, Supplementary Table S2). 

### 3.3. Drug Treatments Received

**Figure 3:**
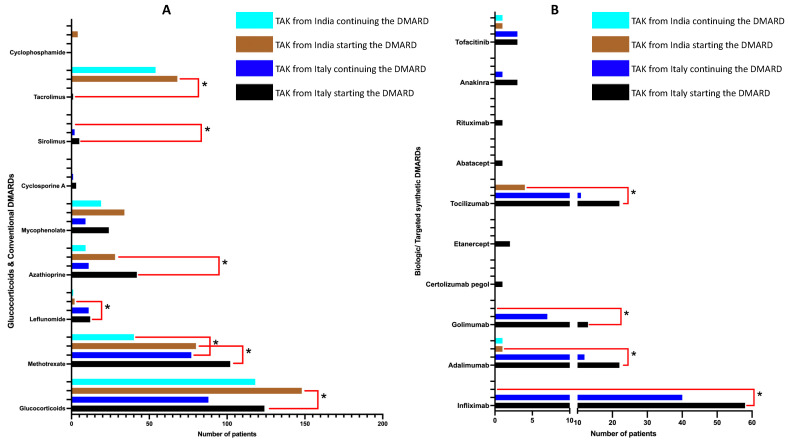
**Use of** (**A**) **conventional DMARDs and** (**B**) **biologic or targeted synthetic DMARDs in patients with Takayasu arteritis from Italy and India.** For each DMARD, black bar represents the number of patients from Italy who had used that DMARD, and blue bar represents the number of patients from Italy who were continuing that DMARD at the last recorded follow-up. Brown bar represents the number of patients from India who had used that DMARD, and cyan bar represents the number of patients from India who were continuing that DMARD at the last recorded follow-up. Those comparisons marked with an asterisk were different between the two cohorts for proportions of patients who had been started on an individual DMARD with *p* < 0.05. DMARD—Disease-modifying anti-rheumatic drug.

**Figure 4:**
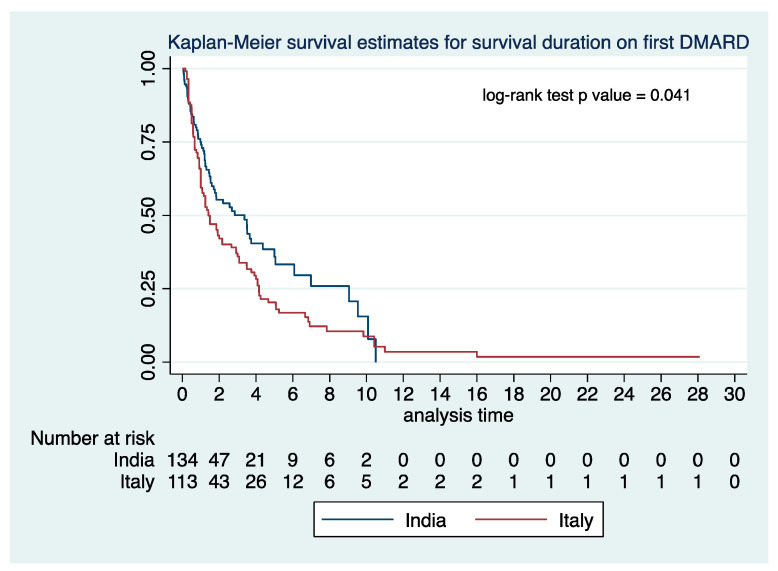
Survival on first DMARD in patients with Takayasu arteritis from Italy and India. DMARD—Disease-modifying anti-rheumatic drug.

A greater number of TAK patients in the cohort from Italy than from India had been started on glucocorticoids (oral as well as intravenous methylprednisolone pulses), with a higher starting dose (mean ± standard deviation, 48.1 ± 13.3 mg vs. 33.2 ± 14.6 mg daily prednisolone equivalent) and for a longer duration (103 vs. 38 months) (Supplementary Table S3). A similar reduction in mean daily prednisolone dose from baseline until the last follow-up was observed in both cohorts. Overall, TAK patients from Italy received a higher mean number of csDMARDs and b/tsDMARDs than from India. Methotrexate, leflunomide, azathioprine, mycophenolate, and tacrolimus had been used in TAK patients from both cohorts. Cyclosporine and sirolimus had only been used in patients from Italy, whereas cyclophosphamide had only been used in patients from India. The use of methotrexate, leflunomide, and azathioprine was greater in TAK patients from Italy, whereas tacrolimus had been used more frequently in TAK patients from India (Figure 3A, Supplementary Table S3). Only five patients from India had been treated with bDMARDs (tocilizumab, *n* = 4, adalimumab, *n* = 1) and one with tsDMARDs (tofacitinib). The use of bDMARDs and tsDMARDs was much more frequent in TAK from Italy. Infliximab, adalimumab, and tocilizumab were the most frequently used bDMARDs, whereas tofacitinib had been used in three patients (Figure 3B, Supplementary Table S3). The persistence on the first DMARD was longer for the cohort from Italy than from India [median (interquartile range) duration for Italy 15.2 (7.6–46.2) months (*n* = 113), for India 13.9 (4.1–39.2) months (*n* = 134), hazard ratio using Cox regression 0.72, 95% CI 0.52–0.99, log-rank test *p*-value 0.041, Figure 4)]. Regarding reasons for a change of first line DMARDs, the switch of one DMARD to another or suspension of DMARD was more common in India, whereas the add-on of DMARDs was more common in Italy (Supplementary Table S4). Methotrexate was the most frequent first-line choice of DMARD in both cohorts. Infliximab was the most frequent second line DMARD in the cohort from Italy, whereas mycophenolate was the commonest second-line DMARD in India. (Supplementary Table S5). In line with the observation of more frequently observed hypertension from India, TAK patients from India had greater usage of anti-hypertensives (both frequency and mean number of anti-hypertensive medications per patient) at baseline. A higher proportion of patients from Italy had received aspirin or statins at baseline (Supplementary Table S3). 

### 3.4. Procedures and Infections on Follow-Up

Thirty-eight percent of patients with TAK from Italy had undergone procedures related to TAK, as opposed to 19% from India (*p*-value for Chi-square test <0.001). A greater number of total procedures in the cohort from Italy (*n* = 111) than from India (*n* = 48) was also recorded. A considerably larger proportion of procedures from Italy (42/111) had been performed before diagnosis when compared with those from India (5/42, *p*-value for Chi-square test <0.001). Duration of disease at the time of the procedure (for those undergoing procedures after diagnosis) was longer for TAK patients from Italy than from India. 

A higher number of serious infections (i.e., requiring hospitalization or resulting in death) was recorded from India than from Italy, although the proportions of patients developing such infections were similar in both cohorts. A similar proportion of patients from the two cohorts was on glucocorticoids and at a similar dose at the time of infection. The proportion of patients on csDMARDs during episodes of serious infection was similar, whereas more patients from Italy were on bDMARDs (Table 2). 

## 4. Discussion

The present comparative analysis of two large cohorts of TAK from distinct geographic regions identified interesting differences in the presentation, diagnostic modalities used, and choices of treatment. 

A considerably greater proportion of patients with TAK from India were males when compared to those from Italy. The Indian cohort was also younger. The disparity in gender between cohorts of TAK from Asian and European or North American populations has been noted before. About 10–15% of TAK cohorts from Europe or North America comprise males when compared with nearly 20–25% from Asian cohorts of TAK [34,35,36,37,38,39,40]. Similarly, younger age at diagnosis has been noted for cohorts from Asia than from Europe or North America [34,35,36,37,38,39,40]. Both cohorts had a similar delay to diagnosis from the first symptom. Previous cohorts of TAK have revealed heterogeneity in the delay to diagnosis, some with shorter (median 0.8 years from France, 1.3 years from Canada, 2.3 years from the United States of America, mean 1.58 years from China) [35,37,38,41], and other with longer diagnostic delays (mean 7.6 years from China) [42]. The frequencies of hypertension, renal failure, and renal artery stenosis were much higher in TAK from India than in Italy. Patients with TAK from Europe and North America [38,43,44,45] tend to have a lesser frequency of hypertension or renal artery involvement than those from Asia [39,40]. Our study also identified a greater prevalence of coronary artery involvement in TAK from Italy than from India. Previous studies have also revealed a greater prevalence of coronary artery involvement in TAK from Europe (9.8% amongst 82 patients with TAK from France) [34] than from Asia (3.6% amongst 1056 TAK from China) [46]. 

Significant disparities in the use of imaging modalities at initial assessment were observed. CDUS was more commonly used in TAK patients from Italy and CT angiography was more frequent in TAK from India. Despite greater radiation exposure, CT angiography might be a more preferred modality in India because it is easier to perform in terms of technical expertise and is more widely available than PET-CT. Moreover, the competing interests for healthcare resources with more common infectious diseases such as tuberculosis limit the available time for radiologists for imaging the arterial system, which might result in such preferences. PET-CT also appeared to be more frequently used in TAK from Italy than from India, although the differences were not statistically significant. However, nearly a third of patients from the Indian cohort had also undergone PET-CT. The functional annotation of hypermetabolic vascular foci with PET-CT makes this an attractive modality for disease activity assessment in TAK. With the increasing availability of PET-CT in India, this imaging modality is likely to be utilized more for the evaluation of TAK. It must be noted that PET-CT often identifies a proportion of active vascular disease in TAK where the CRP is normal [8,47]. A recent survey of international experts in TAK suggested the feasibility of the use of PET-CT as a screening modality for active disease before the recruitment of patients in clinical trials of TAK [48]. The increasing use of PET for LVV disease activity assessment in lesser economically developed settings such as India shall hopefully enable the wider inclusivity of patients in future clinical trials of TAK. Considerable technical expertise is required for CDUS for large vessel imaging. In Italy, the greater prevalence of GCA (where CDUS is often used) when compared with India might have reflected in the greater use of this modality in patients with TAK as well [2,49]. 

The use of glucocorticoids at a greater dose and for longer periods was observed in patients with TAK from Italy than from India. This might have been due to a greater proportion of patients with active disease as well as the longer duration of follow-up of patients with TAK from Italy. Also, the cohort from Italy had a greater prevalence of concomitant autoimmune diseases, which might have necessitated glucocorticoids for their treatment. The prevalent literature suggests similar outcomes in TAK from India treated with a lower or higher dose of glucocorticoids, which might have resulted in the use of a lower dose of glucocorticoids in the cohort from India [50]. Considerably greater use of bDMARDs and tsDMARDs was observed in the TAK cohort from Italy; however, the percentage reduction in glucocorticoids from baseline was similar for both cohorts. Differences in the healthcare systems of Italy and India might explain such differences in patterns of medication use. Nationalized healthcare in Italy (as opposed to considerable out-of-pocket expenses for specialist healthcare in India) might have resulted in greater accessibility to, and consequently the greater use of, bDMARDs and tsDMARDs in Italy [51,52]. Although DEI.TAK and ITAS2010 were higher at baseline for the cohort from India, the relevance of this is difficult to interpret. DEI.TAK and ITAS2010 do not have universally accepted cut-offs for disease activity [53]. Hypertension can be scored up to 2 on the DEI.TAK and 3 on the ITAS2010. A far greater proportion of TAK from India had hypertension than those from Italy, which might have influenced the disease activity scores to some extent. Also, hypertension noted at a particular visit need not necessarily be due to active disease. Rather, it could just reflect the impact of missing the morning dose of anti-hypertensive on the day of the clinic visit, irregular drug compliance, patient anxiety during the present visit (white coat hypertension), or vascular damage [2,54,55,56]. Outcomes on follow-up of these two cohorts might enable a more granular understanding of the impact of differences in quantitative estimates of disease activity scores between the two cohorts. In this context, it is important to reiterate the observation that mortality in the cohort from India was higher than that from Italy, despite a considerably longer duration of follow-up in the latter cohort. 

Amongst csDMARDs, the use of methotrexate, leflunomide, or azathioprine was more frequent in Italy, whereas tacrolimus had been used more frequently in TAK from India. Such disparities in the choice of conventional DMARDs between the two cohorts might relate to the lack of recommendations for the treatment of TAK for the preferential use of a particular csDMARD in TAK [28]. The lack of such recommendations for a specific DMARD relates to the failure of any DMARD (conventional, biologic, or targeted synthetic) to be effective in clinical trials involving patients with TAK [10]. Of note, no such recommendations exist for India or other Asian countries. The lack of accessibility of biologics in India (due to costs as well as heightened risk of opportunistic infections such as tuberculosis) might have resulted in the use of tacrolimus as a csDMARD in a considerable proportion of TAK patients from India. Such use has been extrapolated from the use of tacrolimus in treatment-refractory rheumatoid arthritis or systemic lupus erythematosus in studies from Asia [57,58]. Limited in-vitro evidence suggests the potential utility of tacrolimus as a DMARD in TAK [59]. Interestingly, despite the considerably greater use of bDMARDs and tsDMARDs in patients with TAK from Italy than from India, the overall proportions of patients developing infections were similar. A greater prevalence of bDMARD or tsDMARD use preceding infection episodes from the TAK cohort from Italy than from India possibly relates to the overall greater use of bDMARDs in the cohort from Italy. 

A greater proportion of TAK from Italy had undergone disease-related procedures than from India. A possible explanation for this was the considerably longer duration of follow-up in the cohort from Italy. A considerably larger proportion of these procedures predated the diagnosis of TAK in the cohort from Italy than from India (possibly before referral to the rheumatologist). This might relate to greater accessibility to endovascular procedures in Italy than in India (where interventional radiology is still a developing specialty) [60,61,62]. General practitioners should be educated that vascular procedures should only be done during periods of inactive TAK; otherwise they portend a greater risk of peri-operative morbidity or mortality [63]. 

To the best of our knowledge, this is the first comparative analysis of patients with TAK from two distinct geographic regions with different socioeconomic realities. A large number of patients for what is a rare form of large vessel vasculitis was a strength of the study. The retrospective nature of the study resulted in missing information for some data points from the cohort from India; however, missing information was only for a small proportion of the dataset (generally <5%). Information that was not recorded in the clinic files might have been missed, which is a limitation of the design of a retrospective chart review. While some data on follow-up of both cohorts has been presented (deaths, medical therapies used, comparison of duration of survival on the first DMARD, procedures undertaken related to TAK, and infections observed), a more detailed analysis of follow-up data, including that on clinical disease activity measures, was beyond the scope of the present study. Such follow-up data, preferably collected prospectively, shall enable a better understanding of whether the differences in diagnostic and treatment modalities affect patient outcomes. The present cohorts were hospital-based rather than community-based cohorts; therefore, drawing estimates of the incidence and prevalence of TAK is beyond their scope. It must also be kept in mind that the two cohorts were based on single vasculitis clinics from Italy and India. The analyses from both cohorts might not represent the national practices of the two countries but rather reflect the prevalent practices at two established vasculitis clinics. The observed differences in the diagnostic methods and treatments between the two cohorts could have been due to different clinical practices in these two regions, partly influenced by availability of recommendations for the management of TAK from Europe but not from Asia [28,49]. The analysis of Human leucocyte antigen (HLA) or other genetic associations was also beyond the scope of the present study [64].

## 5. Conclusions

Understanding disparities in the presentation, diagnosis, and treatment of less common diseases like TAK from different parts of the world might facilitate the development of suitable practice guidelines for diagnosis and management which apply to a wider patient demographic. The lack of a quality evidence base to guide the medical management of TAK is a hindrance to developing evidence-based recommendations for TAK, instead relying predominantly on expert opinion. Randomized controlled trials of investigational therapies in TAK, whenever planned in the future, should attempt to include patients from diverse geographic regions to enable better generalizability of their results. 

## Figures and Tables

**Table 1:** Characteristics of the cohorts.

	Italy (*n* = 127)	India (*n* = 191)	*p* Value *	*p* Values Corrected for Multiple Testing
**Demographic characteristics**				
Age at cohort entry Mean (± SD)	35.9 (±13.79)	29.8 (±11.3)	**<0.001**	-
Sex distribution (Female: Male)	110:17	142:49	**0.005** ^a^	-
Diagnostic delay (years) Mean (± SD)	3.4 (±4.6)	3.0 (±4.0) (*n* = 189)	0.413	-
Duration of follow-up (months) Mean (± SD)	130 (±103)	46 (±51)	**<0.001**	-
**Prevalence of recorded comorbidities at the initial presentation**				
Diabetes mellitus	4 (3.1%)	6 (3.1%)	>0.999 ^b^	>0.999
Smoking	34 (26.8%)	2 (1.1%)	**<0.001** ^b^	**<0.001**
Dyslipidemia	8 (6.3%)	0 (0%)	<0.001 ^b^	**<0.001**
Cancer	1 (0.8%)	0 (0%)	0.399 ^b^	0.9529
Other autoimmune diseases	20 (15.7%)	3 (1.6%)	**<0.001** ^b^	**<0.001**
Specify list with numbers	Sarcoidosis (*n* = 3) Inflammatory bowel disease (*n* = 6; ulcerative colitis—3; Crohn’s disease—2; indeterminate colitis—1) Psoriasis (*n* = 6) Psoriatic arthritis (*n* = 2) Uveitis (*n* = 2) Relapsing polychondritis (*n* = 2) Extravascular IgG4-Related disease (*n* = 1) Spondyloarthritis (*n* = 1)	Spondyloarthritis (*n* = 1) Systemic sclerosis (*n* = 1) Inflammatory bowel disease (*n* = 1—Crohn’s disease)	-	-
Other comorbidities	1 (0.7%)	13 (6.8%)	**0.010** ^b^	0.061
Specify list with numbers	HIV (*n* = 1)	Hypothyroidism (*n* = 10) Rheumatic heart disease (*n* = 1) Epilepsy (*n* = 1) Osteoporosis (*n* = 1)	-	
**Disease activity and outcomes**				
DEI.TAK score at first visit [mean (± SD)]	5.4 (±3.5)	9.4 (±6.3)	**<0.001**	-
ITAS2010 at first visit [mean (± SD)]	6.9 (±4.9)	11.0 (±7.3)	**<0.001**	-
Proportion with active disease at first visit	116 (91.3%)	153 (80.1%)	**0.007**	-
Mortality (*n*, %)	1 (0.8%)	10 (5.2%)	0.055 ^b^	-

* Unpaired t-test for mean (SD), Chi squared ^a^/Fisher’s exact ^b^ for proportions. DEI.TAK—Disease Extent Index in Takayasu arteritis; ITAS2010—Indian Takayasu arteritis Clinical Activity Score; HIV—Human immunodeficiency virus; SD—Standard deviation. *p* values < 0.05 are highlighted in bold.

**Table 2:** Infections requiring hospitalization or resulting in death.

	Italy (*n* = 127)	India (*n* = 191)	*p* Value *	*p* Value Corrected for Multiple Testing
Number of Infections (in total)	16	36	-	-
Number of patients developing infections (*n*, % of cohort)	12 (9)	30 (16)	0.106 ^a^	0.429
How many were on prednisolone (*n*, % of episodes)	11 (69)	32 (89)	0.113 ^b^	0.451
Infection episodes while on conventional DMARDs (*n*, % of episodes)	9 (56)	28 (78)	0.114 ^a^	0.454
Infection episodes while on biologic or targeted synthetic DMARDs (*n*, % of episodes)	8 (50)	1 (3)	**<0.001** ^b^	**<0.001**
Dose of prednisolone at time of infection (mg/day, mean ± SD)	9.8 ± 7	14.3 ± 10.8	0.205	0.682
DMARD at time of infection—list out (*n*, %)	Methotrexate + Infliximab (*n* = 1) Methotrexate + Golimumab (*n* = 1) Infliximab (*n* = 1) Methotrexate (*n* = 3) Golimumab (*n* = 1) Azathioprine (*n* = 1) Sirolimus + Tocilizumab (*n* = 1) Sirolimus + Golimumab (*n* = 1) Sirolimus + Tofacitinib (*n* = 1) Mycophenolate (*n* = 1) Anakinra (*n* = 1)	Mycophenolate + Tocilizumab (*n* = 1) Tacrolimus + Methotrexate (*n* = 1) Methotrexate (*n* = 11) Azathioprine (*n* = 6) Tacrolimus (*n* = 4) Mycophenolate (*n* = 4) Cyclophosphamide (*n* = 1)	-	

* Unpaired t-test for mean (SD), Chi squared ^a^/Fisher’s exact ^b^ for proportions. DMARD—Disease-modifying anti-rheumatic drug; SD—Standard deviation. *p* values < 0.05 are highlighted in bold.

## Data Availability

All the analyses performed for this article have been reported in the main text or in the supplementary files. Data pertaining to the article shall be shared on reasonable request to the corresponding author (Corrado Campochiaro, campochiaro.corrado@hsr.it).

## Supplementary Materials

The following supporting information can be downloaded at: https://www.mdpi.com/article/10.3390/diagnostics12123102/s1, Table S1: Clinical features at presentation; Table S2: Imaging modalities and vascular involvement at initial assessment; Table S3: Treatments received; Table S4: Reasons for failure of disease-modifying anti-rheumatic drugs; Table S5: Choice of first and second-line disease-modifying anti-rheumatic drugs.
