# Supplementary material for: Impact of Geographic Location on Diagnosis and Initial Management of Takayasu Arteritis: A Tale of Two Cohorts from Italy and India
Source: Diagnostics (Basel). 2022 Dec 9;12(12):3102. doi: 10.3390/diagnostics12123102 (PMC9777621; doi:10.3390/diagnostics12123102)
Supplement: Supplementary file 1 [file diagnostics-12-03102-s001.zip › diagnostics-1988869-supplementary.pdf]

Supplementary Table S1: Clinical features at presentation$

	Italy (n=127)	India (n=191)	Odd's ratio (95% CI) (Italy vs India)	p value* corrected for multiple testing
**Clinical features at presentation [n(%)]**				
None	4 (3)	0 (0)	-	0.451[b]
Seizure	0 (0)	9 (5)	-	0.266[b]
Documented TAK retinopathy	0 (0)	2 (1)	-	>0.999[b]
Aortic regurgitation	0 (0)	11 (6)	-	0.090[b]
Acute coronary syndrome	7 (5)	0 (0)	-	**0.035**[b]
Other	17 (13)	22 (12)	-	-
Specify list with numbers	Dyspnoea (n=9)<br>Flank pain (n=1)<br>Cardiac arrest (n=1)<br>Myocarditis (n=1)<br>Lung infarction (n=2)<br>Pulmonary arterial hypertension (n=1)<br>Digital necrosis (n=1)	Palpitations (n=8)<br>Digital necrosis (n=1)<br>Hemoptysis (n=2)<br>Breathlessness (n=9)<br>Oral ulcers (n=1)<br>Occasional upper limb numbness (n=1)		
**Baseline investigations**				
ESR (mm/hr) [mean (± SD)]	61 ± 32	46 ± 32 (n=186)	-	**<0.001**
CRP (mg/L) [mean (± SD)]	37 ± 42	24 ± 32 (n=179)	-	**0.007**
Hemoglobin (g/dL) [mean (± SD)]	11.8 ± 1.2	11.4 ± 2.0 (n=185)	-	0.128

$Those clinical features other than the ones depicted in Figure 1 are presented here.
* Chi squared[a]/ Fisher's exact[b] for proportions
BP – Blood pressure; 95% CI – 95% confidence interval; CRP – C-reactive protein; ESR – Erythrocyte sedimentation rate; SD – Standard deviation; TAK – Takayasu arteritis; TIA – Transient ischemic attack.
NA – Not assessable
p values <0.05 are highlighted in bold

Supplementary Table S2: Imaging modalities and vascular involvement at initial assessment

	Italy [n(%)]	India [n(%)]	Odd's ratio (95% CI) (Italy vs India)	p value corrected for multiple testing
**Imaging modalities[$]**	(n=127)	(n=191)		
PET-MRI	1 (0.8)	0 (0)	-	0.953[b]
**Vascular involvement**	n=127	n=190		
Coronary	15 (12)	3 (2)	8.35 (2.49 – 27.52)	**0.005**[b]
Right subclavian	65 (51)	75 (39)	1.61 (1.02 – 2.55)	0.590[a]
Left subclavian	81 (64)	138 (73)	0.66 (0.41 – 1.08)	0.888[a]
Right carotid	74 (58)	75 (39)	2.14 (1.36 – 3.34)	**0.022**[a]
Left carotid	79 (62)	99 (52)	1.51 (0.96 – 2.36)	0.823[a]
Right vertebral	22 (17)	6 (3)	6.43 (2.52 – 15.55)	**<0.001**[a]
Left vertebral	18 (14)	22 (12)	1.26 (0.64 – 2.38)	>0.999[a]
Pulmonary	21 (17)	11 (6)	3.22 (1.46 – 7.21)	**0.040**[a]
Brachiocephalic	34 (27)	48 (25)	1.08 (0.64 – 1.79	>0.999[a]
Ascending aorta	36 (28)	36 (19)	1.69 (1.01 – 2.84)	0.679[a]
Arch of aorta	44 (35)	67 (35)	0.97 (0.61 – 1.54)	>0.999[a]
Descending thoracic aorta	45 (35)	94 (49)	0.56 (0.36 – 0.89)	0.259[a]
Abdominal aorta	57 (45)	105 (55)	0.66 (0.42 – 1.03)	0.797[a]
Celiac trunk	38 (30)	49 (26)	1.23 (0.75 – 1.99)	>0.999[a]
Superior mesenteric artery	36 (28)	44 (23)	1.31 (0.78 – 2.19)	>0.999[a]

Inferior mesenteric artery	4 (3)	8 (4)	0.74 (0.24 – 2.40)	>0.999[b]
Right renal	27 (21)	81 (43)	0.36 (0.22 – 0.61)	**0.002**[a]
Left renal	25 (20)	78 (41)	0.35 (0.21 – 0.60)	**0.002**[a]
Right iliac	21 (16)	12 (6)	2.94 (1.37 – 6.15)	0.074[a]
Left iliac	21 (16)	13 (7)	2.70 (1.29 – 5.37)	0.129[a]
Right femoral	4 (3)	2 (1)	3.06 (0.70 – 16.21)	0.996[b]
Left femoral	2 (2)	3 (2)	0.99 (0.18 – 4.94)	>0.999[b]

[$]Those imaging modalities other than the ones depicted in Figure 2A are presented here.

* Chi squared[a]/ Fisher's exact[b] for proportions

95% CI – 95% confidence interval; C+ - Coronary involvement; CT – Computerized tomography; PET – Positron emission tomography; MRI – Magnetic resonance imaging; NA – Not assessable; P+ - Pulmonary involvement

p values <0.05 are highlighted in bold

Supplementary Table S3: Treatments received

	Italy (n=127)	India (n=191)	p value*
**Glucocorticoids**			
n(%)	124 (98)	148 (77)	**<0.001**[b]
on intravenous methylprednisolone n(%)	7 (5)	2 (1)	**0.033**[b]
Starting dose (mean with SD)	48.1 ± 13.3	33.2 ± 14.6 (n=143)	**<0.001**
Continuing at last follow-up n(%)	88 (71)	118 (80)	0.093[a]
Duration in months (mean ± SD)	103 ± 90	38 ± 36 (n=140)	**<0.001**
Percentage reduction in prednisolone at last visit	90 ± 14	87 ± 17 (n=119)	0.134
**Methotrexate**			
n(%)	102 (80)	80 (42)	**<0.001**[a]
Continuing at last follow-up n(%)	77 (75)	40 (50%)	**<0.001**[a]
Duration in months (mean ± SD)	57 ± 58	33 ± 32 (n=77)	**0.001**
**Leflunomide**			
n(%)	12 (9)	2 (1)	**<0.001**[b]
Continuing at last follow-up n(%)	11 (92)	1 (50%)	0.275[b]
Duration in months (mean ± SD)	13 ± 7	8 ± 7 (n=2)	0.368
**Azathioprine**			
n(%)	42 (33)	28 (15)	**<0.001**[a]
Continuing at last follow-up n(%)	11 (26)	9 (32)	0.589[a]
Duration in months (mean ± SD)	55 ± 55	31 ± 34 (n=26)	0.050
**Mycophenolate**			
n (%)	24 (19)	34 (18)	0.804[a]
Continuing at last follow-up n(%)	9 (38)	19 (56)	0.168[a]
Duration in months (mean ± SD)	31 ± 37	17 ± 17 (n=34)	0.057
**Cyclosporine A**			
n(%)	3 (2)	0	0.063[b]

Continuing at last follow-up n(%)	1 (33)	0	-
Duration in months (mean ± SD)	26 (19)	0	-
**Sirolimus**			
n(%)	5 (4)	0	0.001[b]
Continuing at last follow-up n(%)	2 (40)	0	-
Duration in months (mean ± SD)	80 (64)	0	-
**Tacrolimus**			
n(%)	1 (0.8)	68 (36)	**<0.001**[b]
Continuing at last follow-up n(%)	0 (0)	54 (79)	0.217[b]
Duration in months (mean ± SD)	22	18 ± 20 (n=68)	-
**Cyclophosphamide**			
n(%)	0	4 (2)	0.153
Continuing at last follow-up n(%)	0	0 (0)	-
Duration in months (mean ± SD)	0	6 ± 2 (n=4)	-
**Total number of csDMARDs received** (mean with SD)	1.5 ± 0.9	1.1 ± 0.9	**<0.001**
**Infliximab**			
n(%)	58 (46)	0	**<0.001**[b]
Continuing at last follow-up n(%)	40 (69)	0	-
Duration in months (mean ± SD)	58 ± 51	0	
**Adalimumab**			
n(%)	22 (17)	1 (0.5)	**<0.001**[b]
Continuing at last follow-up n(%)	12 (55)	1 (100%)	0.999[b]
Duration in months (mean ± SD)	32 ± 31	10	-
**Golimumab**			
n(%)	13 (10)	0	**<0.001**[b]
Continuing at last follow-up n(%)	7 (54)	0	-
Duration in months (mean ± SD)	21 ± 21	0	-

**Certolizumab pegol**			
n(%)	1 (0.8)	0	0.399[b]
Continuing at last follow-up n(%)	0 (0)	0	-
Duration in months (mean ± SD)	5	0	-
**Etanercept**			
n(%)	2 (2)	0	0.159[b]
Continuing at last follow-up n(%)	0 (0)	0	-
Duration in months (mean ± SD)	14 ± 3	0	-
**Tocilizumab**			
n(%)	22 (17)	4 (2)	**<0.001**[b]
Continuing at last follow-up n(%)	11 (50)	0 (0)	0.113[b]
Duration in months (mean ± SD)	30 ± 32	10 ± 11 (n=4)	0.235
**Abatacept**			
n(%)	1 (0.8)	0	0.399[b]
Continuing at last follow-up n(%)	0 (0)	0	-
Duration in months (mean ± SD)	19	0	-
**Rituximab**			
n(%)	1 (0.8)	0	0.399[b]
Continuing at last follow-up n(%)	0 (0)	0	-
Duration in months (mean ± SD)	3	0	-
**Anakinra**			
n(%)	3 (2)	0	0.063[b]
Continuing at last follow-up n(%)	1 (33)	0	-
Duration in months (mean ± SD)	11 ± 3	0	-
**Tofacitinib**			
n(%)	3 (2)	1 (0.5)	0.306[b]
Continuing at last follow-up n(%)	3 (100)	1 (100%)	>0.999[b]
Duration in months (mean ± SD)	28 ± 9	12	-

**Baricitinib**			
n(%)	0	0	-
Continuing at last follow-up n(%)	0	0	-
Duration in months (mean ± SD)	0	0	-
**Total number of ts or bDMARDs received** (mean with SD)	1 ± 1.2	0.04 ± 0.21	**<0.001**
**Antihypertensives**			
**n(%)**	56 (44)	148 (77)	**<0.001**[a]
Mean (± SD) number of antihypertensives at presentation	0.9 ± 1.2	1.7 ± 1.4	**<0.001**
**Aspirin**	71 (56)	50 (26)	**<0.001**[a]
**Clopidogrel**	9 (7)	21 (11)	0.243
**Statin**	43 (34)	23 (12)	**<0.001**[a]

* Chi squared[a]/ Fisher's exact[b] for proportions
SD – Standard deviation
p values <0.05 are highlighted in bold

Supplementary Table S4: Reasons for failure of disease-modifying anti-rheumatic drugs

Reason for failure	Italy (n=95)	India (n=66)	p value* corrected for multiple testing
Switch to another DMARD	26 (27%)	31 (47%)	**0.031**
Suspension of DMARD	12 (13%)	19 (29%)	**0.031**
Add-on of another DMARD	57 (60%)	16 (24%)	**<0.001**

DMARD – Disease-modifying anti-rheumatic drug
*Chi square test
p values <0.05 are highlighted in bold

Supplementary Table S5: Choice of first and second-line disease-modifying anti-rheumatic drugs

	**Italy (n=114)**	**India (n=143)**
First-line DMARD	n= 114<br><br>Methotrexate (85)<br>Azathioprine (20)<br>Leflunomide (1)<br>Mycophenolate (6)<br>Infliximab (2)	n=143<br><br>Methotrexate (64)<br>Tacrolimus (50)<br>Azathioprine (12)<br>Mycophenolate (12)<br>Cyclophosphamide followed by Azathioprine (2)<br>Cyclophosphamide (1)<br>Tocilizumab (1)<br>Methotrexate + Mycophenolate (1)
Second-line DMARD (switch or add-on)	n=81<br><br>Infliximab (41)<br>Azathioprine (9)<br>Methotrexate (8)<br>Golimumab (6)<br>Adalimumab (6)<br>Tocilizumab (5)<br>Leflunomide (2)<br>Sirolimus (2)<br>Tofacitinib (1)<br>Mycophenolate (1)	n=50<br><br>Mycophenolate (15)<br>Azathioprine (11)<br>Methotrexate (10)<br>Tacrolimus (8)<br>Leflunomide (2)<br>Tocilizumab (2)<br>Adalimumab (1)<br>Cyclophosphamide (1)

DMARD – Disease-modifying anti-rheumatic drug
